# Colorectal cancer screening in Semarang, Indonesia: A multicenter primary health care based study

**DOI:** 10.1371/journal.pone.0279570

**Published:** 2023-01-03

**Authors:** Hery Djagat Purnomo, Cecilia Oktaria Permatadewi, Agung Prasetyo, Didik Indiarso, Hesti Triwahyu Hutami, Dik Puspasari, Devia Eka Listiana, Suhartono, Herna Rizkia Armatussolikha, Suryanto Setyo Priyadi, Sri Sadono, Silvina, Nurhayati, Samsudin, Ahnaf, Muhammad Hidayanto, Puriyanto Wahyu Nugroho, Nur Dian Rakhmawati, Agus Susanto, Mukti Setiawan, Mochamad Sonny

**Affiliations:** 1 Faculty of Medicine Diponegoro University, Division of Gastroenterohepatology, Department of Internal Medicine, dr Kariadi Hospital, Semarang, Indonesia; 2 Faculty of Medicine Diponegoro University, Division of Anatomic Pathology, Department of Internal Medicine, dr Kariadi Hospital, Semarang, Indonesia; 3 Faculty of Public Health Diponegoro University, Department of Environmental Health, Semarang, Indonesia; 4 Faculty of Medicine Diponegoro University, Magister Program of Biomedical Science, Semarang, Indonesia; 5 Bangetayu Primary Health Care, Semarang, Indonesia; 6 Pegandan Primary Health Care, Semarang, Indonesia; 7 Kagok Primary Health Care, Semarang, Indonesia; 8 Bandarharjo Primary Health Care, Semarang, Indonesia; 9 Bugangan Primary Health Care, Semarang, Indonesia; 10 Ngesrep Primary Health Care, Semarang, Indonesia; 11 Srondol Primary Health Care, Semarang, Indonesia; 12 Tlogosari Kulon Primary Health Care, Semarang, Indonesia; 13 Pandanaran Primary Health Care, Semarang, Indonesia; 14 Rowosari Primary Health Care, Semarang, Indonesia; University of Toronto, CANADA

## Abstract

Colorectal cancer (CRC) is a major public health problem in Indonesia. It ranks among the top four cancers with high mortality rates. CRC screening is expected to improve early diagnosis that can reduce mortality and morbidity rate. Primary health care-based CRC screening in Indonesia has not yet been performed. This study was conducted to obtain information about prevalence, adenoma detection rate and public compliance for CRC screening in Semarang, Indonesia. This cross-sectional study was done across 10 primary health care centers in Semarang during April—October 2021. The screening method used Immunochromatography Faecal Occult Blood Tests (I-FOBT) as the primary test. Patients with positive I-FOBT result would be referred to Kariadi hospital for colonoscopy and histology examination. A total of 221 patients were included, 66.1% were female, mean age was 59.38 ± 7.48 years. Participation rate was 63%, 37 patients (16.7%) were I-FOBT positive, 26 patients (70.27%) underwent colonoscopy. Colonoscopy compliance rate was 70.27%. The colonoscopy results were haemorrhoid (30.8%), polyps (30.8%), malignancy (19.2%), colitis (7.7%), diverticulosis (7.7%), and normal (3.8%). The adenoma detection rate was 26.9%. BMI abnormality (overweight and obese) (OR 10.968; 95% CI 2.33–51.55) and family history of malignancy (OR 18.800; 95% CI 5.13–68.85) increased the risk of colorectal cancer and adenoma and respectively. The prevalence of I-FOBT positive in primary health care centers is high. The CRC screening program based on primary care should be considered. Public awareness education should be considered to increase colonoscopy compliance.

## Background

Colorectal cancer (CRC) has become a major concern worldwide. In 2020, it was estimated that 1.9 million cases of colorectal cancer caused 0.9 million deaths. Currently, CRC is the third most diagnosed cancer in men and the second most diagnosed in women [1]. This situation also occurs in Indonesia, where CRC is a growing health problem and included in the top three cancers with the highest mortality rate. According to GLOBOCAN, the incidence of CRC in Indonesia ranked second in men and fourth in both men and women, among other types of cancer. CRC has a sharp increase in the number of incidents with 12.8 per 100,000 adult populations, and have a 9.5% mortality rate of all cancer cases [2]. Currently, an enormous treatment cost burden (approximately 18.6% of the total financing covered by national health insurance in Indonesia) comes from catastrophic diseases. CRC with high morbidity and mortality certainly plays a significant role [3].

Early studies of CRC in Indonesia during 2008–2012 in 26 cities and 14 provinces showed that colorectal cancer was higher in men (54%) than in women (46%). Most cases occurred at the age of 50–54 years [4]. Currently, there is a shift in the age trend in cases of CRC, which begins to develop at a younger age. A European study found a significant increase in CRC before the age of 50. The highest increase occurred in the age group 20–29 years with 7.9% per year. It was followed by the age group 30–39 years with 3.4% and the age group 40–49 years with an increase of 1.6% [5]. Most guidelines recommend starting colorectal cancer screening in average-risk individuals at the age 50 years [6–9]. In 2018, the American Cancer Society recommended starting screening at age 45 [9]. This recommendation is based on the severity of the disease, increased incidence in younger subjects, and the assumption that screening in the 45 to 49 year age group will have a better significant preventive effect than those aged 50 years and over [10].

CRC mortality is highly dependent on the stage when it was found; the earlier, the better prognosis. The Surveillance, Epidemiology, and End Results (SEER) Program stated that only 37.5% of cases of CRC were diagnosed at a localized stage (stage I), and 5-year relative survival for localized colorectal cancer was 90.6% [11]. In 2017, there was a national consensus on recommendations for CRC screening in Indonesia [12]. However, it has not been implemented and covered by national health insurance. So data regarding CRC screening in Indonesia is still lacking. In addition, public awareness regarding malignancy screening (including CRC) for early detection is still low [13]. Colorectal cancer screening is expected to improve early diagnosis so that curative efforts can be made, reduce mortality morbidity, and lower the cost [14]. A new strategy is needed to increase community participation in implementing CRC screening. We involved a primary health care (PHC) or “Puskesmas” (Pusat Kesehatan Masyarakat) to increase public participation. Primary health care in Indonesia has a vital role in prevention, screening, and dealing with various reasons for individual or community health problems [15].

A primary care based study for CRC Screening in Indonesia has not been performed. We report the first study involving primary health care in a multicenters manner to implement colorectal cancer screening in Indonesia. This research was conducted to determine the prevalence, adenoma detection rate, and public compliance regarding CRC screening in Semarang, Indonesia. This data then can be used as an evidence for developing CRC screening strategies in Indonesia. It is suggested that the government establish a national CRC screening program covered by the national health insurance to expand the community participation. Detecting early-stage CRC is expected to reduce the economic burden and morbidity due to advanced CRC.

## Methods

### Study design and ethical approval

This is a primary health care based colorectal cancer screening program in average-risk population conducted from April to October 2021 in over 10 Primary Health Care in Semarang, Central Java, Indonesia. Average-risk individuals are people aged 45 years and older, with no history of colorectal cancer or inflammatory bowel disease, no family history with colorectal cancer. The target population were men and women aged over 45 years who visited Primary Health Care. Exclusion criteria from this study were history of taking anticoagulant drugs, recent blood in stool, colorectal cancer, gastrointestinal polyps and patients who died during the study. Clinical characteristics and risk factors (age, gender, education level, body mass index, and past medical history) were recorded. Each participant provided written, informed consent prior to participation in this study, and this study was authorized in advance by the institutional review board of the Research Ethics Committee of the Faculty of Medicine, Diponegoro University. The ethical clearance was given by Dr. Kariadi Hospital (790/EC/KEPK RSDK/2021).

### Sampling and populations

A total of 10 PHCs were chosen from 37 health centers using convenience sampling with consideration of locations (both central and peripheral districts), proportion of patient visits, and the availability of human resources. The participants were randomly selected from patients who were waiting in line for medical purposes or just to accompany relatives. Following informed consent and history-taking, we provided a container for stool samples to subjects who agreed to be screened, and the samples were returned the next day for inspection. I-FOBT was used as the primary test. The faeces were examined using an R40-112 I-FOBT device, which can detect hemoglobin at 10 ng/ml. If the result was positive, the subject was invited for a colonoscopy as a secondary test followed by a histology examination.

### Data analysis

Data analysis were done using the statistical software package SPSS (IBM Corp., New York; version 25). Validation processes were done to ensure the integrity of recorded data. The I-FOBT as primary test was performed by international standard-compliant laboratory. Colonoscopy was performed by a competent gastroenterologist, and histological examination was conducted by two anatomical pathologists to validate biopsy results. Demographic characteristics were expressed as number and percentage. Differences associated factors between groups were compared using the χ2 test—including sex (male and female), age (categorised into 45–50, 50–60, > 60 years) educational background (low: primary school or below primary to high school; high: high school or above), history of diabetes mellitus, smokers, polyp, inflammatory bowel disease, and family history of CRC. Odd Ratios (ORs) and their 95% Confidence Intervals (CIs) were presented. Colonoscopy results were quantified, specifically for adenoma (pre-cancerous stage) and malignancy. Additionally, in order to evaluate our screening quality; the polyp detection rate (PDR), adenoma detection rate (ADR), and participation rate were calculated as follows:

PDR = (number of examinations with polyps/total number of colonoscopy procedure) × 100%

ADR = (number of examinations with adenomas/total number of colonoscopy procedure) × 100%

Participation Rate = (number of participants who returned stool samples for analysis/ total number of patients who were given study explanation and accept a stool sample container)

## Result

### Characteristic of participants

A total 221 subjects from 10 Primary Health Care among Semarang City met our criteria. The distributions of participants from each PHC were recorded in Table 1. Mean age was 59.38 ± 7.48 SD years with the largest group distribution was > 60 years (50.7%) The demographic characteristic of this study is shown in Table 2.

**Table 1:** The distribution of primary screening participation.

Primary Healthcare	Number of Participant (N)
Kagok	50
Pandanaran	36
Bandarharjo	28
Tlogosari Kulon	27
Bugangan	21
Rowosari	19
Pegandan	12
Ngesrep	11
Srondol	11
Bangetayu	6
**Total**	221

**Table 2:** Demographic characteristic.

Variable	N	Percentage (%)
**Sex**		
Male	75	33.9
Female	146	66.1
**Age Group**		
< 50 year	33	14.9
50–60 year	76	34.4
>60 year	112	50.7
**Body Mass Index**		
Normal	141	63.8
Overweight	46	20.8
Obese	34	15.4
**Education Level**		
Low	86	38.9
High	135	61.1
**Medical History**		
Diabetes Mellitus	86	38.9
Alcohol consumption	0	0
Smokers	7	3.2
Polyp	0	0
IBD	0	0
History of taking OAINS	27	12.2
Previous colonoscopy history	0	0
Family history of other malignancy	2	0.9

The most common comorbidity found was diabetes mellitus with 38.9% (Table 2).

### Primary screening positivity

A total of 350 subjects were offered to participate in the screening program and received a container for stool samples. However only 221 participants return the sample container to the PHC next day. It showed the participation rate of 63%. From 221 subjects with average risk of CRC, 37 of them had positive I-FOBT result. The distribution was shown in Table 3. The prevalence of positive I-FOBT result among average risk for CRC in this study population was 16.7%.

**Table 3:** Positive I-FOBT results.

Variable	CRC Screening Result (I-FOBT)			
	Positive		Negative	
	N	%	N	%
**Sex**				
Male	13	17.3	62	82.7
Female	24	16.4	122	83.6
**Age**				
<50 year	6	18.2	27	81.8
≥50 year	31	16.5	157	83.5
**BMI**				
Abnormal (over-obese)	19	26.4	53	73.6
Normal	7	5.1	131	94.9
**Medical history**				
**Diabetes Mellitus (DM)**				
Yes	12	14.0	74	86.0
No	25	18.5	110	81.5
**Smoking**				
Yes	2	28.6	5	71.4
No	35	16.4	179	83.6
**NSAID consumption**				
Yes	16	59.3	11	40.7
No	21	10.8	173	89.2
**Family History of Other Malignancy**				
Yes	18	81.8	4	18.2
No	19	9.5	180	90.5

### Colonoscopy results, adenoma and polyp detection rate

Subjects with a positive I-FOBT result were referred for a colonoscopy procedure. Among 37 subjects with positive I-FOBT results, 11 subjects refused due to fear from the procedure (n = 7), and the Covid-19 pandemic (n = 4). The colonoscopy compliance rate in this study was 70.27% (26/37). The grouping of results are shown in Table 4.

**Table 4:** Colonoscopy results.

Colonoscopy result	N	Percentage (%)
Haemorrhoid	8	30.8
Colitis	2	7.7
Polyp	8	30.8
Malignancy	5	19.2
Diverticulosis	2	7.7
Normal	1	3.8

**Fig 1:**
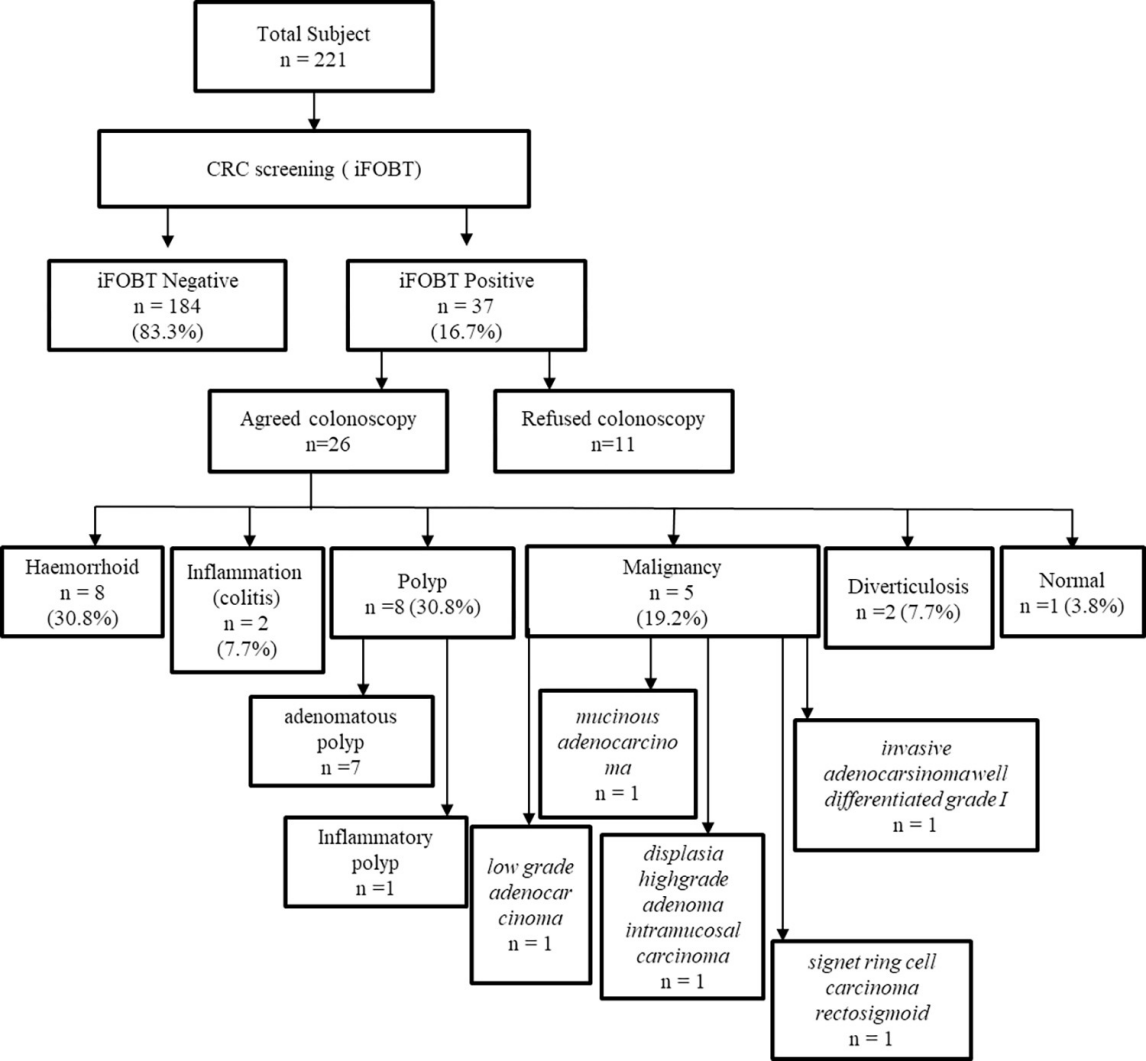
Study flow chart, screening results and followed up examination.

The colonoscopy findings were 8 subjects had haemorrhoids, 2 subjects had colitis, 8 subjects had polyps, 5 subjects had malignancy, 2 subjects had diverticulosis, and 1 subject with normal results. The polyp detection rate (PDR) was 30.8% (8/26) and adenoma detection rate (ADR) of 26.9% (7/26) as shown in Fig 1. There were two type of polyps found: adenomatous polyp (n = 7) and inflammatory polyp (n = 1). Meanwhile, the histological patterns of malignancy were low grade adenocarcinoma (n = 1), mucinous adenocarcinoma (n = 1), dysplasia high-grade adenoma intramucosal carcinoma (n = 1), signet ring cell carcinoma rectosigmoid (1), invasive adenocarcinoma well differentiated (n = 1).

### Factors associated with pre-cancerous and cancerous lesion

We performed bivariate analysis to assess factors associated with pre-cancerous (adenoma/polyp adenoma) and cancerous lesion in 210 subjects. The result was shown in Table 5.

**Table 5:** Factors associated with pre-cancerous and cancerous lesion.

Variable	Colonoscopy Finding				p-value	OR (95% CI)
	Adenoma/CRC		Non malignancy			
	N	%	N	%		
**Sex**						
Male	3	4.2	69	95.8	0.485	0.623 (1.63–2.37)
Female	9	6.5	129	93.5		
**Age**						
≥50 years	9	5.1	168	94.9	0.363	0.536 (0.13–2.09)
<50 years	3	3.1	30	90.9		
**Education level**						
Low	8	6.3	120	93.7	0.676	1.300 (0.37–4.46)
High	4	4.9	78	95.1		
**BMI**						
Abnormal (over-obese)	10	13.9	62	86.1	<0.001	10.968 (2.33–51.55)
Normal	2	1.4	136	98.6		
**Diabetes Mellitus (DM)**						
Yes	4	4.9	77	95.1	0.701	0.786 (0.23–2.70)
No	8	6.2	121	93.8		
**Smoking**						
Yes	0	0.0	5	100	0.577	0
No	12	16.4	193	83.6		
**NSAID consumption**						
Yes	3	13.6	19	86.4	0.091	3.140 (0.78–12.60)
No	9	4.8	179	95.2		
**Family History of Other Malignancy**						
Yes	6	37.5	10	62.5	<0.001	18.800 (5.13–68.85)
No	6	9.5	188	90.5		

* significant, p < 0.05.

BMI (OR 10.968; 95% CI 2.33–51.55) and family history of other malignancy (OR 18.800; 95% CI 5.13–68.85) may be associated with precancerous and cancerous lesion in colonoscopy results and histology examination (p value < 0.05). However further information and larger study is needed to ensure BMI and family history of other malignancy are risk factors for CRC.

## Discussion

### Optimizing CRC screening in primary care

We evaluated individuals with an average risk of CRC based on recommendations for CRC screening. CRC screening in Indonesia have not been fully implemented due to various reasons such as lack of knowledge and community participation, screening tools, and costs which are not covered by the national health insurance [16]. This study was designed for average-risk population. One of the screening tests for colorectal cancer is Faecal Occult Blood Test (FOBT) in particular immunochromatography FOBT [17]. I-FOBT was considered a novel method and has never been used widely as screening method for CRC in Indonesia. One of the CRC screening test used in Indonesia is the Guaiac-based Occult Blood Test (G-FOBT) [13]. Unfortunately, G-FOBT in Indonesia is still cannot accessed by primary health care directly, or can only be accessed in commercial clinical laboratories. I-FOBT has better sensitivity and specificity than G-FOBT. The G-FOBT has high false positive because of interaction with several foods that create false positive result, so it requires diet restriction. Examining patient using I-FOBT does not require diet restriction and more specific in detecting bleeding in the colon and rectum [10].

As shown in Table 2, there were 221 qualified participants recruited in this study. Based on the number of pots at the beginning of the study (350), it was found that the participation rate in this study was 63%. It is higher than previous epidemiological studies in general populations, which is only 40% [13]. Previous study in 2012 by Koo et al., among 14 country in Asia-Pacific region showed that Indonesia had low participation test (3%) compared by other countries [18]. In 2016, we also conducted a population-based study of 500 subjects (unpublished, see supporting information), it was found that the participation rate of the subjects who agreed to be screened using the I-FOBT was only 51% (n = 257). Each PHC’s participation in this study was uneven. This was probably caused by several reasons, such as location differences (fewer participants in suburban areas; for example, there were only 6 participants in Bangetayu PHC), the proportion of patient visits, and other factors (demographic, socio-economic, and education level), but no further analysis was conducted in this study. However, the involvement of primary healthcare contributed to an increase of screening participation rates.

The largest age group distribution of subjects was > 60 years (50.7%) with a mean age of 59.38 years. This is because of most patients visit primary health services in Indonesia to check their chronic diseases such as hypertension or type 2 diabetes mellitus, which are more common in the elderly (> 50 years) [19]. Diabetes mellitus is the most common disease among the subjects (38.9%).

### Screening evaluation

Positive results of I-FOBT were obtained in 37 (16.7%) subjects and dominated by women (64.8%). Our research is in line with a previous study conducted by Abdullah, et al on 278 subjects spread over five health care facilities in Depok, West Java, Indonesia. The prevalence of a positive I-FOBT test in the asymptomatic population is 4%, dominated by women (72.7%) [13]. The reason why CRC is more common in women is probably due to their higher awareness rate than men [20]. Most of the subjects had a high level of education (61.1%), mostly graduated from high school. However, many subjects refused to be screened for various reasons. Their little knowledge about colorectal cancer symptoms and its risk factors becomes a barrier to CRC screening in Indonesia [21].

The polyp detection rate in this study was 30.8% and adenoma detection rate of 26.9%. Adenoma detection rate (ADR) is a quality indicator for colonoscopy. It is defined as the proportion of patients who had at least one colorectal adenoma detected during a colonoscopy procedure performed by an endoscopist [22,23]. The PDR and ADR rates obtained in this study are lower, when compared to ADR recommendation for colonoscopy screening in CRC with ≥ 25% (men ≥ 30%, women ≥ 20%); but similar to the figures of some Asian countries [24]. A large colonoscopy series in Korea reported ADR of 32.8% [25]. Japan reported ADR of 26.7% [26]. However, the ADR in the average-risk population mostly tends to be lower when compared to the high-risk population and the surveillance setting [27,28]. Colorectal polyps can be divided into neoplastic, non-neoplastic, and sub-mucosal polyps. One type of neoplastic polyp is adenomatous and hyperplastic polyps, the most common colorectal polyps with 80–90% prevalence. Adenomatous polyps potentially become malignant due to a process known as "adenocarcinoma sequencing". Polyp size, the severity of dysplasia, and histopathological examination results are associated with an increased risk of malignancy [29].

### Risk factors for CRC

The analysis showed that BMI and family history of other cancers may associate with colorectal cancer. A person with a family history of other malignancy has a higher risk of developing colorectal cancer [30]. Recent meta-analyses show a significant correlation in the incidence of CRC with family history of CRC in a first-degree relative [31]. Wilkinson’s study showed that individuals who had a first-degree relative (FDR) with CRC or advanced adenoma, diagnosed at any age, should undergo a colonoscopy every 5 to 10 years from age 40 to 50. Alternatively, ten years younger than the age at diagnosis of FDR, stool immunochemical testing at intervals of every 1 to 2 years could also be performed [32]. A previous study from Swedish Family-Cancer Database among 1.8 million cancer patients and over 200,000 CRC cases showed consistent familial associations of CRC with other Cancer [33]. However, studies about the association of CRC with a family history of other malignancies are still very limited and need further research.

Consistent with our finding, recent epidemiological study suggested a strong positive correlation between obesity and colorectal cancer [34,35]. A meta-analysis showed around 11% of colorectal cancer (CRC) cases had been attributed to overweight and obesity in Europe and associated with increased risk of colon cancer in men by 30–70%, whereas the association was less consistent in women [36]. A recent cohort study of Asian women in Singapore observed that obesity has a positive association with risk of colon cancer but not risk of rectal cancer [37]. Indeed, several studies find the association between overweight or obesity and CRC risk on both gender [38–40]. Obesity is associated with CRC through various aspects, including nutriology, adipokines and hormones, inflammation, gut microbiota, and bile acids [41]. Further studies focusing on the incidence of CRC in the overweight/obese population of Indonesia are highly recommended.

## Study limitation

This study was a regional-level, has not involved the entire PHC in Semarang and cannot represent the national prevalence of CRC. The distribution of participants remain uneven, there are PHCs with small number of participants. Therefore the results cannot be generalized to all population in Semarang. Further study with a larger scope including all PHC in Semarang and more public participation is highly recommended.

We also recommend to conduct a special session for general practitioners and public counseling in PHC regarding the urgency of Primary Care Based CRC screening before the study. Consistent follow-up and mentoring also required to boost PHC participation, particularly among PHC with a small number of participation.

The distribution of the age range in the population was uneven, most of the participants were elderly. This study uses a cross-sectional design where the variables are simultaneously measured, the outcome and the exposure cannot be determined because both are examined at the same time. It would be better if the study were conducted in a cohort to monitor each patient’s progress.

## Conclusion

The prevalence of I-FOBT positive in primary care is relatively high with adenoma detection rate of 26.9%. This study shows that primary health care based CRC screening improving the public participation rate. The CRC screening program based on a primary care should be widely implemented in Indonesia. Moreover, further public educational program are necessary to increase screening participation and colonoscopy compliance.

## Supporting information

S1 File Unpublished work. Preliminary study.
(PDF)

S2 File Dataset. (XLSX)
