# Supplementary material for: Colorectal cancer screening in Semarang, Indonesia: A multicenter primary health care based study
Source: PLoS One. 2023 Jan 3;18(1):e0279570. doi: 10.1371/journal.pone.0279570 (PMC9810157; doi:10.1371/journal.pone.0279570)
Supplement: S1 File — Preliminary study. (PDF) [file pone.0279570.s001.pdf]

Colorectal cancer screening in Banyumanik, Semarang, Indonesia (A Population Base Study)

Hery Djagat Purnomo, MD, PhD
Division of Gastroenterology Hepatology - Departement of Internal Medicine, Dr. Kariadi Hospital, Faculty of Medicine University of Diponegoro, Semarang, Indonesia

Presented in : Korean International Digestive Endoscopy Congress 2019

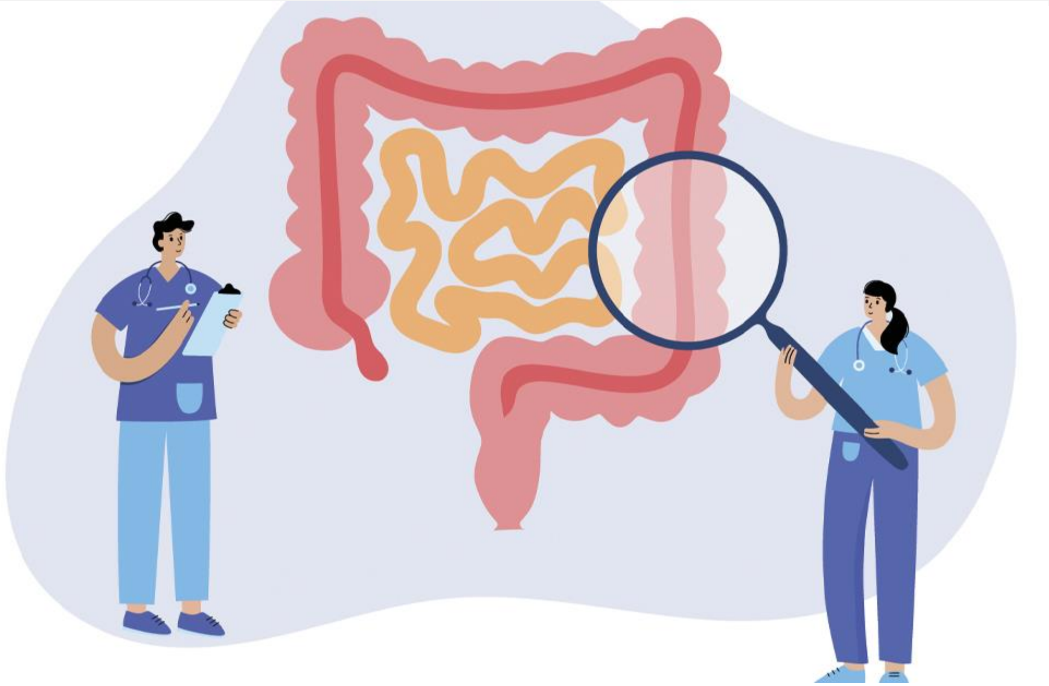

Population based screening

500 people invited to do colorectal cancer screening with **FOBT**

Main Goal of CRC Screening in Kelurahan Ngesrep , Kecamatan Banyumanik, Semarang, 2016

- Increasing **people's awareness** about colorectal cancer
- Increasing the **scope of colorectal cancer screening**
- Obtaining **health profile**, especially colorectal cancer, in Kelurahan Ngesrep, Kecamatan , Banyumanik ,Semarang
- Creating a **baseline for Colorectal Prevention and Screening Program**

CRC Screening Method in Semarang 2016

- **Population Target**
  - **500 asymptomatic patients** (35-75 years old)
- **Screening Method**
  - **FIT, Colonoscopy**
- **Executor**
  - Dinas Kesehatan Kota Semarang and government district (Kecamatan Banyumanik and Kantor Kelurahan Ngesrep Semarang)
  - Primary Healthcare Office Ngesrep Banyumanik
  - Indonesia Association Gastrointestinal and Endoscopy society Semarang
  - Dr Kariadi Hospital, Division GastroenteroHepatology Dep Internal medicine, Semarang
  - Private laboratorium (Lab Prodia) in Semarang

CRC Screening Semarang 2016

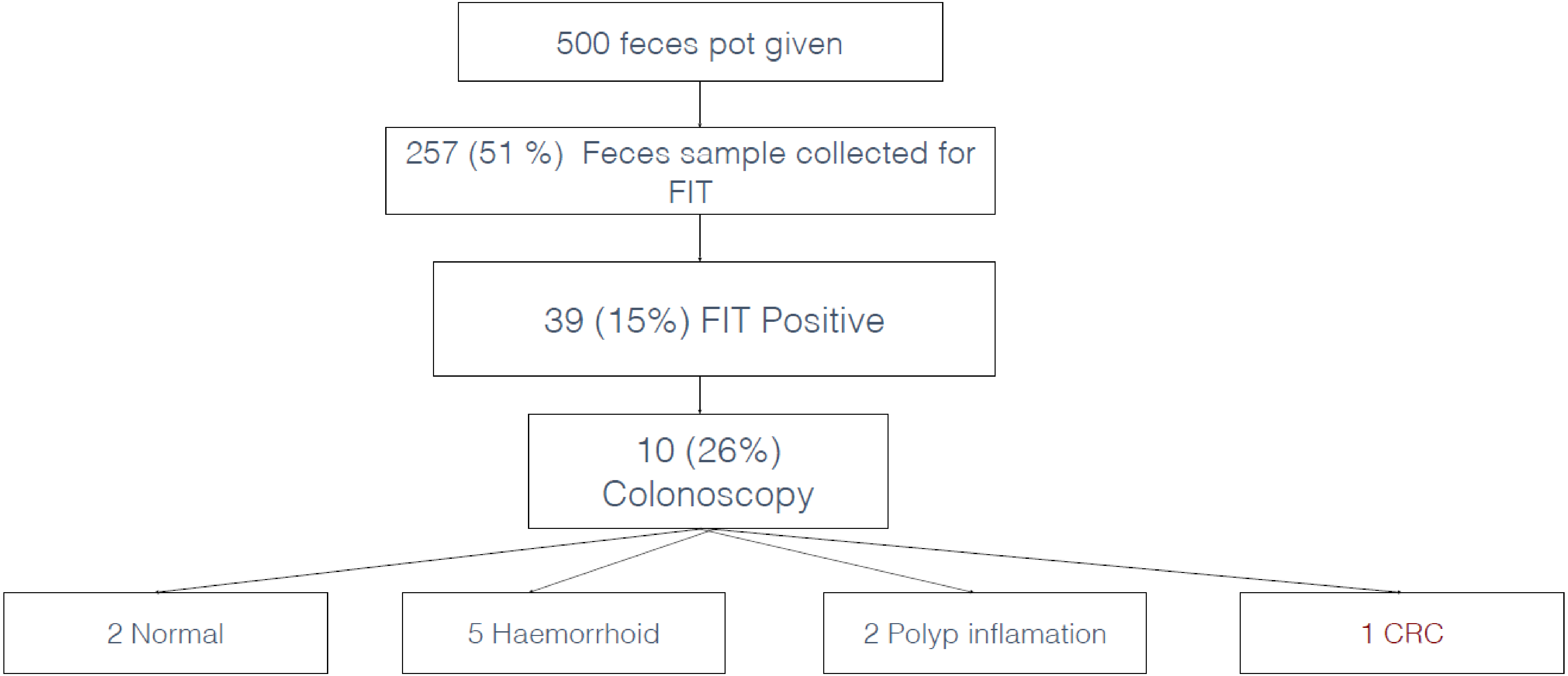

Hery Djagat P, et al , Konas PGI-PEGI-PPHI, Semarang report 2016
